# Optimal Res-UNET architecture with deep supervision for tumor segmentation

**DOI:** 10.3389/fmed.2025.1593016

**Published:** 2025-05-30

**Authors:** Rahman Maqsood, Fazeel Abid, Jawad Rasheed, Onur Osman, Shtwai Alsubai

**Affiliations:** ^1^Department of Information Systems, University of Management and Technology, Lahore, Pakistan; ^2^Department of Computer Science and Information Technology, University of Lahore, Lahore, Pakistan; ^3^Department of Computer Engineering, Istanbul Sabahattin Zaim University, Istanbul, Türkiye; ^4^Department of Software Engineering, Istanbul Nisantasi University, Istanbul, Türkiye; ^5^Applied Science Research Center, Applied Science Private University, Amman, Jordan; ^6^Department of Electrical and Electronics Engineering, Istanbul Topkapi University, Istanbul, Türkiye; ^7^Department of Computer Science, College of Computer Engineering and Sciences, Prince Sattam Bin Abdulaziz University, Al-Kharj, Saudi Arabia

**Keywords:** magnetic resonance imaging, Residual U-Net, deep supervision, medical image analysis, dice loss, image segmentation challenges, encoder-decoder networks, attention mechanism

## Abstract

**Background:**

Brain tumor segmentation is critical in medical imaging due to its significance in accurate diagnosis and treatment planning. Deep learning (DL) methods, particularly the U-Net architecture, have demonstrated considerable promise. However, optimizing U-Net variants to enhance performance and computational efficiency remains challenging.

**Objective:**

To develop an optimized Residual U-Net (Res-UNET) architecture enhanced by deep supervision techniques to improve segmentation accuracy of brain tumors on MRI datasets, specifically addressing challenges of conventional segmentation methods.

**Methods:**

The study implemented a detailed evaluation of multiple U-Net variations, including basic U-Net, Res-UNet with Autoencoder regularization, and attention-enhanced U-Net architectures. Training was conducted using the BraTS 2018 public MRI dataset. Deep supervision was integrated to improve gradient propagation and segmentation accuracy. The model employed a Dice loss combined with focal loss to handle data imbalance effectively. The proposed network was evaluated using extensive ablation studies, examining the effects of encoder complexity, convolutional filter count, and strategic post-processing.

**Results:**

The proposed Res-UNET with deep supervision outperformed other variants, achieving an average Dice score of 0.9498 through five-fold cross-validation. Post-processing strategies improved the robustness of segmentation, particularly enhancing the accuracy of small tumor regions. Comparatively, conventional U-Net architectures yielded lower Dice scores and required significantly longer training times. The study indicates the benefit of integrating deep supervision and residual connections for enhanced model performance.

**Conclusion:**

Optimized Res-UNET with deep supervision significantly enhances segmentation accuracy for brain tumors in MRI images, surpassing traditional U-Net models. This model addresses critical issues such as dataset imbalance, lack of annotated data, and computational inefficiencies. Future studies should consider the broader application of optimized U-Net variants across other medical imaging segmentation tasks.

## 1 Introduction

Biomedical and human intelligence breakthroughs over the last several years have addressed many ailments, yet cancer remains a significant challenge due to its unpredictable nature. This disease continues to pose a considerable threat to humanity. Brain tumor malignancy is one of the most devastating new illnesses (1, 2). In 2015, there were around 23,000 cases of brain tumor malignancy in the United States. About 36.3 percent (29,320 cases) were meningiomas; 26.5 percent (21,200 cases) were gliomas; about 16.2 percent (13,210 cases) were pituitary tumors; and the remainder of the cases were other forms of brain tumors, such as malignant, medulloblastoma, and lymphomas (3, 4). Many factors contribute to cancer-related sickness and morbidity, but these are the main ones. Timely and accurate diagnosis of this disease is essential for effective management and prompt patient intervention.

Grade 1: It’s safe to say that they are benign tumors since the cells they contain look like the brain’s regular cells.

Grade 2: These may be benign or cancerous and seem somewhat different from normal cells.

Grade 3: These seem significantly different from conventional cells and are highly harmful to them.

Grade 4: The tumor is growing and spreading at an alarming rate. Cells of this kind have an unusual appearance, and they are dangerous.

**Figure 1:**
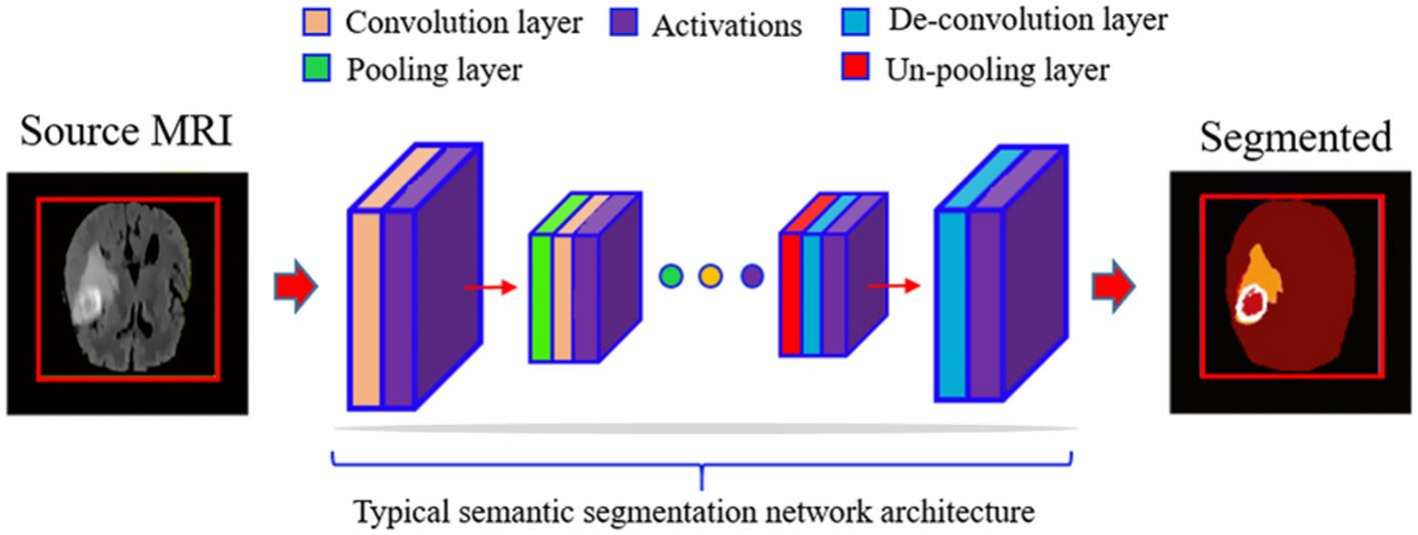
Illustration a standard deep learning segmentation process with convolutional neural networks (CNN). The process begins with compression (encoder) of raw MRI images through convolution, activation, and pooling, followed by a decoder that expands the latent representation to achieve segmentation during inference. The condensed latent depictions are further extended using inverse techniques. Training is kept up-to-date on the network from beginning to finish. When testing, the segmentation labels are used in a forward pass.

Medical microscopy is becoming increasingly important in everyday medical diagnostics research as a result of the advancements in modern optics. So, data from medical imaging tests must be studied. Brain tumors have been a major focus of medical study because of their prevalence and complexity (5). Image data analysis of images of brain tumors is commonly used to diagnose brain cancers. To accurately assess a patient’s status, looking at images of brain tumors is necessary. Image findings may be inaccurately interpreted due to the accretion of clinicians’ personal medical information, variances in experience, and visual tiredness (6). It is possible to get information about the shape, size, and location of human tissues and organs without ionizing radiation using MRI (Magnetic Resonance Imaging) (7). A range of imaging techniques and strategies have been utilized for the diagnosis and treatment of a brain tumor. MRI images are segmented to isolate the affected area of brain tissue using image processing methods like segmentation. A conventional DL (Deep Learning) segmentation procedure begins with a compression of the raw images using many layers of convolution, activation, and pooling in a fully connected CNN (Convolutional Neural Network) (see Figure 1). As a management tool, automated segmentation methods help trace borders between distinct tissue sections with varying degrees of automation and allow automated volumetric pathological MRI signal interpretation.

DL (Deep Learning) has played a crucial role in the present rise of AI in almost every industry (8). Computer vision, natural language processing, particle physics, DNA analysis, and research on brain circuits are only a few examples of the wide variety of domains where it has recently made significant advances. Medical imaging researchers have also shown great interest in this field recently. Computers can learn complex mathematical data representation models, which can subsequently be utilized for accurate data analysis, using the DL framework.

**Figure 2:**
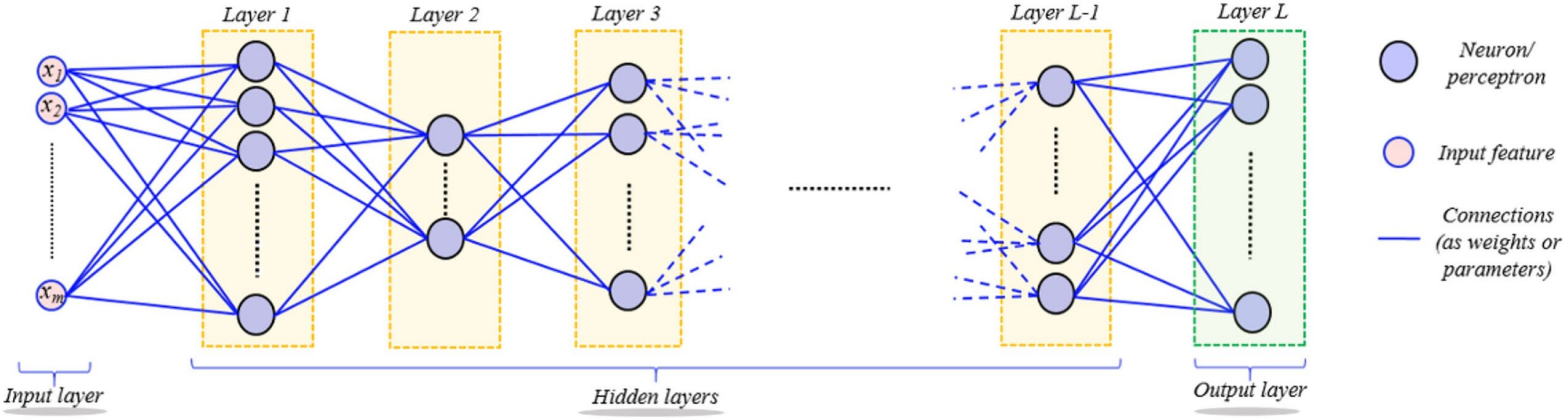
Depiction of the hierarchical layered structure of a deep neural network (DNN). Neurons or perceptron layers process input signals hierarchically through linear and nonlinear transformations, showing how deep networks learn complex representations from input data.

**Figure 3:**
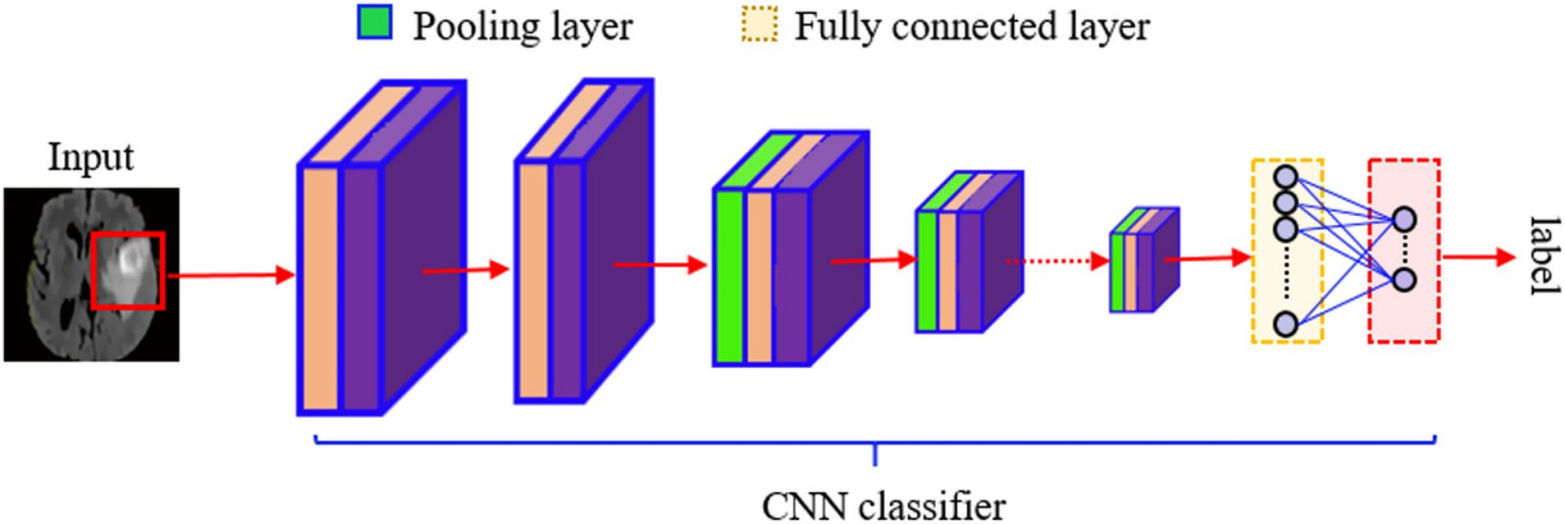
Flow diagram showing a CNN-based classifier for detecting brain tumors. The input image passes through several convolutional and pooling layers, which are then fully connected layers to output the final configuration label.

Hierarchical models produce non-linear and/or linear functions of incoming data, weighted by model parameters. An overarching goal of data-driven techniques is to utilize training data collection to improve the computer model’s performance, e.g., classification, by learning the model’s parameters progressively. ANN (Artificial Neural Network) is a learning algorithm that consists of several basic computational blocks (neuron/perceptron layers), while its structures (network weights) define the forte of interconnections between layers. A DNN (Deep Neural Network) (8) comprises numerous layers of neurons and perceptrons coupled in an inter-layer form as shown in Figure 2. It is possible for the DL models to successfully execute the same job using unseen data (testing data) beforehand, after being trained for it (9). DL’s ability to forecast better than other ML algorithms makes it stand out. A back-propagation approach is used to learn the parameters of a deep model, allowing some variants of the standard GD (Gradient Descent) method to achieve the chosen feature values recursively. Only an epoch of model learning occurs when all training data is used to adjust the model’s parameters. Many current deep-learning models can be used for hundreds of epochs of training. The difficulty hampers diagnostic image processing in identifying attributes during pattern recognition (10). Figure 3 depicts the brain tumor segmentation’s crucial feature extraction and selection phases.

The remainder of the article is structured as follows: Section 2 discusses how current limitations in brain tumor detection experiments may impact the entire model, including multi-classification and segmentation. Section III focuses on the majority of the material studied. Section 4 offers insights and experimental results on future directions. The experimental steps are as follows: For the BraTS21 challenge, we conducted comprehensive ablation studies to identify the optimal U-Net version and training strategy. For U-Net variations, we tested U-Net alongside Res-UNet, utilizing deep supervision and various loss functions. Our framework has increased the encoder complexity and the number of CN filters. Section 5 concludes the article.

## 2 Existing challenges

This section outlines some of the biggest obstacles to using DL in medical image analysis. Table 1 lists significant challenges faced in deep learning for medical image analysis.

**Table 1:** A comparative overview highlighting significant challenges faced in deep learning for medical image analysis, with detailed descriptions clarifying the nature of each issue.

Sr. No	Challenges	Description
1	Lack of appropriately annotated datasets	We can only generalize an architecture with a high no. of features if we use a vast quantity of data to conclude the hyperparameters.
2	Imbalance data	Although many samples are inconclusive, only a few positive results may be used in a dataset to retrain an algorithm to identify brain cancers.
3	Lack of confidence interval	Only a single reliability coefficient may be deduced from the output waveform of a neuron.
4	Difficulty in understanding medicinal task CNN	Identify the primary tasks with significant annotation material and the desired outcome.
5	Covering deep features for imaging tasks	Techniques that need fewer training samples for natural images
6	Training partially frozen deep networks	It has been possible to overcome the shortage of annotated large-scale datasets by training partially frozen networks to perform medical imaging.
7	Inter learning with heterogeneous deep modeling	At which input vector could be controlled for each task, yet the tasks are interconnected?
8	Creating simulated data utilizing GANS	Regarding medical imaging, the GAN structure should be utilized with extreme care. Keep in mind that GANs do not learn how images were originally distributed.
9	Variety of matrix factorization methods	Methods are typically not as powerful as advanced techniques, such as using GANs to boost data samples
10	Wide-reaching perspective	Experts do not readily understand medical literature in other areas.

### 2.1 Lack of datasets with appropriate annotations

As the DL model involves complex mathematical functions, it stands out from other Machine Learning algorithms. Generally, as models get more complicated, we add additional layers to the model - i.e., go deeper. For a model to be generalized, we would need to utilize a large quantity of data to determine the values of the parameters. Almost every machine-learning technique relies on this occurrence in some form or another. A sophisticated model built from a small amount of data typically overfits the information used and performs poorly on all subsequent data sets. Learning deep models in sectors where only a limited quantity of training data is available is intrinsically incompatible. PACS (Images Archiving and Communication System) and OIS (Oncology Information System) systems provide a lot of medical images, but they do not have enough annotations to train deep models effectively; thus (11), cannot utilize them to train deep models, either. Aside from that, the annotations required for deep models are often incompatible with standard medical practice. This complicates matters even more for experts who need to incorporate noise-free notes. It is necessary to provide such public datasets for medical imaging to train Deep Learning models on large-scale training datasets.

### 2.2 Imbalanced data

Imbalanced datasets are more prevalent in medical imaging than in conventional computer vision tasks. Only a few positive samples may be included in the dataset when training a model to identify brain tumors, but significant exposure to negative samples is available. Training deep networks with unbalanced knowledge leads to biased models (12). Many diagnostic imaging operations have a low frequency of positive sample occurrences, which makes matching the original data challenging. Consequently, considerable care must be taken while running deep models for diagnostic tasks.

### 2.3 Lake of confidence interval

A neuron’s output signal could only be viewed as a single likelihood; the DL’s recent work refers to a model’s output as “forecast confidence.” The absence of a confidence interval around a projected result is often not desired in medical imaging jobs. End-to-end deep learning approaches are becoming more popular in medical imaging, according to Litjens et al. (13). This problem has hampered Deep Learning’s extensive use in medical imaging.

### 2.4 Difficulty in understanding medicinal task CNN

Medical image analysis may benefit from transfer learning, and it is clear from the research that disentangling medical activities will be helpful in this endeavor. Transfer learning efficiency has recently been boosted by carefully selecting the source and destination domains/tasks (14). As a fundamental premise, disassociation facilitates the determination of source tasks about which reasonably substantial annotated data sets are accessible and the determination of target tasks, whereby the source models may deliver higher effectiveness if fine-tuned. Another method for dealing with less data is investigating transfer learning between medical imaging and natural image activities. The ability to transfer information to current natural image models may substantially impact patient MRI image analysis using DL.

### 2.5 Covering deep features for imaging tasks

According to current research, many medical task models are trained from beginning to end. End-to-end modeling is often more viable for DL in domains with large-scale annotated data. Using strategies that need fewer training samples for real images, a (15) established that parameters obtained from deep models may be utilized to learn more efficient higher-level features. Their deep characteristics were further tied together before being sent into a classifier. If this is the case, deep qualities are used as input samples for the wrapping method.

### 2.6 Training partially frozen DL

An essential concept in ML is that more training data is needed to develop increasingly complex computational models. The depths of a structure are typically a determinant of its intricacy in deep learning since deeper networks need more features and bigger training datasets. This technique of breaking down pictures from the most basic to the most complex levels of abstraction using neural networks for image processing is called CNN. Early CNN layers educate them to apply comparable filters to a range of natural pictures, which is well-known. According to this, by freezing a few layers upon layers in a network, feature values for a collection of pictures that are likely to be similar, we may minimize the number of learning parameters in a network. Similar models learned on similar tasks may copy parameter values directly. The absence of annotated massive datasets could be mitigated by learning partly frozen networks for diagnostic imaging.

### 2.7 Inter learning with heterogeneous deep modeling

When it comes to Computer Vision online contests, it’s not uncommon for there to be a lack of adequate instruction. Such tournaments tend to have the same winning methods. Rather than learning a single DL model, numerous deep models are trained and integrated to determine the final results. Network topologies (16), complexity, and loss functions are all considered while developing models to use the best data supplied. For example, the joint logit layer, or computing losses while accounting for all networks by integrating them at the output layer, are two methods used to merge several networks. Multi-task learning is also recognized to be efficient in the context of deep learning when training data is sparse. This way, models for several tasks may be learned using just a subset of the training data, but the tasks are still connected. Because of this, the data annotations of several jobs are intertwined. However, learning to do many things at once might be more difficult than learning to do one thing at a time.

### 2.8 Creating simulated data utilizing GANS

Currently, GANs (Generative Adversarial Networks) are paying close attention to the Computer Vision group because of the group’s capacity to mimic the distributions from which images are sampled. The GAN system may create synthetic images that seem realistic in any domain. Models trained using just (limited) original domain information often outperform those trained using these images. GANs have a unique attribute of importance to Medical Image Analysis. Additionally, these studies point out several possible issues in this regard and promising opportunities for GANs as a medical image analysis data production technique. For medical imaging, the GAN framework should be used with caution, according to (17). Keeping in mind that GANs mimic the original distribution of images is essential. As a result, the GAN-generated images may look much different from the originals. This means that fine-tuning a model using just the original photos is always preferable to training the final model using data that contains GAN-generated data.

### 2.9 Wide-reaching perspective

We can make a meaningful remark concerning DL research by studying the literature in diverse domains. To put it another way, as a result of advances in related domains, advancement in Deep Learning research has accelerated dramatically (18). Computer Vision literature was the first to establish the concept of ‘residual learning’, which allowed for deep networks. AlphaGo Zero’s tabula rasa algorithm was eventually made practical because of this notion. Medical literature in other fields is difficult for professionals to comprehend.

### 2.10 Variety of matrix factorization methods

It has also been proven that a few simple strategies for supplementing data have improved deep model performance generally in the literature on computer vision and pattern recognition, even though modern strategies, such as employing GANs to enhance data samples, are more powerful, these methods are still worth using.

## 3 Related work

This section summarizes the recent advances in brain tumor segmentation that use deep learning. We concentrate primarily on research articles released after 2017 while examining the most significant contributions from preceding years.

Multifunctional 3D CNN for tumor identification by Li (19) focuses on the poor accuracy outcomes of existing approaches for brain tumor detection. The Multidisciplinary 3D CNN employs a combination of three-dimensional spatial models with differing properties to identify brain lesions. Adding a normalization layer and max-pooling to the networks will help alleviate the issue of overfitting. The lost output may be improved to expand the function learning of the observed space using the weighted loss during activation. As an example of how these techniques may be used to increase what has previously been utilized to detect a tumor by a minimal and vast volume of the lesion space, a replacement loss performance and efficiency can be upgraded to reduce the non-focal space interference. Experiments show that the method aimed to locate tumor lesions with greater correlation coefficients is successful. By evaluating the two-dimensional and single-mode tumor detection approaches, 3D CNN approaches significantly improve detection accuracy.

When Ding uses (20), the findings of using a deep residual network to enhance multi-model tumor pictures in the section are outstanding. The receptive area may be increased without sacrificing resolution because of its ability to overcome the problem of VG. Specific data from areas with small tumors may be overlooked because of the reduction in picture resolution caused by convolutional techniques operating on a single pixel. These factors necessitated the usage of spatial unification blocks to capture just the tumor’s particular location. It analyzes the link between this pixel and the surrounding area to obtain information on the spatial arrangement of brain tumors. This intermediate supervision block also has a pyramid and multi-hierarchical structure that lowers the accumulation of errors during network training. The “boost learning” concept ensures the network can produce more accurate forecasts. Multi-classified loss accounts for the loss of intermediate suggestions in the middle layers and the reduction of the output forecast to achieve the middle supervision effect. Based on the results of our tests, our system can transmit and expand the variety of information necessary to improve the hierarchy of medical picture recognition.

For tumor segmentation, the Dinga (21) network has been found. It is believed that the UNet has been frequently employed in medical image segmentation because of its link with up-sampling processes. Complex medical images do not work correctly—the stacking process results in a disproportionate increase within specific parameters. Choosing between potency and dependability is a bad trade-off. For these reasons, UNet is improving its network design to be more suited for tumor detection. Our blocks, known as the Easy Reduction SMCSRNet (Spatial Multi-scale Content Aware Residual Network), form an SMCSRNet (SRNet) structure. The SRNet’s primary advantage is that the number of parameters is reduced by 4/5 compared to the original UNet. Certain bridge connections are implemented at some point during the down-sampling process. As a bonus, the projected method’s efficiency is higher than the stacked UNet’s. The outcome is virtually as excellent as the most popular Dense Net or ResNet after additional research on other PSNs.

Chen Jie Ge described an improved MRI image dataset for genetic tumor classification by pairwise GAN (22). Glioma datasets currently available include several scans with missing or incomplete data. To deal with the problem of tiny brain tumor datasets and an insufficient picture modality for DL, we adopted a paired Generative Adversarial Network (GAN) model. When used with other imaging modalities, pairwise GANs may produce synthetic MRI pictures (MRIs). The post-processing strategy leverages a majority vote to merge slice-level glioma subtype classification data for a patient-specific diagnosis. It is possible to fine-tune a training strategy for glioma using both GAN-augmented and actual MRIs. The recommended technique was evaluated on a brain tumor dataset for IDH1 mutation and IDH1 wild-type gene variation to classify glioma molecular subtypes. The data has shown encouraging results in research (with an accuracy of 88.82%).

Deep and handcrafted tomography brain imaging alternatives are described by Hasan (23) in an easy-to-understand manner. Classification using a scanner. Magnetic resonance imaging (MRI) brain scans may be classified based on their capacity to extract relevant characteristics, a critical first step in the process. This has led to several research proposals proposing different ways for extracting information from MRI images of the brain that may detect abnormal brain growths. A method known as the MGLCM is also used to find handcrafted characteristics. They exploited SVM to classify significant attributes collected from the MRI images to enhance the MRI brain image classification. This deep learning approach with handcrafted features, paired with an SVM classifier, achieved a classification accuracy of 99.30%.

Jia (24) identifies and categorizes the images using multiple deep learning algorithms for brain tumor image processing. A new automated technique based on structural, morphological, and relaxometry data has been developed to separate the central vascular system from the MRI images. The segmentation characteristic is distinguished by a high degree of homogeneity between the anatomy and the neighboring brain tissue. Hidden node layers are used in ELM, a type of learning algorithm. Prediction and classification networks, for example, are often used in various fields. Tumor identification accuracy in MRI images has been trained and verified using the probabilistic neural network classification technique. Nearly 98.51% of aberrant and normal tissue may be identified using Magnetic Resonance Imaging (MRI).

Liu (25) explains the Deep C-LSTM Neural Network for identifying epileptic seizures and tumors utilizing high-dimensional EEG inputs. Electrical impulses in the human brain may be studied and diagnosed using electroencephalography (EEG), a commonly used tool. The relatively short identifiable EEG portion hampers real-time seizure detection. To identify seizures and malignancies in the human brain, a novel form of deep convolutional long-term memory (C-LSTM) has been developed (open and closed). Predicts results every 0.006 s, with a detection time of only 0.006 s (one second). Deep C-LSTM outperforms DCNNN and LSTM when compared to other deep learning algorithms. The total accuracy attained is more than or equal to 98.80%.

Mallick (26) used deep neural network-based deep wavelet autoencoders (DNN-DWAE) to classify brain images. The image processing technique is frequently employed in the medical profession to enhance the detection and treatment phases. Image compression using the Deep Wavelet Autoencoder (DWA) technology combines the autoencoder’s fundamental feature reduction function with the wavelet transform’s image decomposition property. The use of DNN for additional classification tasks significantly reduces the feature set size when these two methods are combined. Consideration was given to the proposed DWA-DNN image extractor. The DWA-DNN classifier’s performance indicators were compared to those of existing classifiers, including Autoencoder-DNN or DNN.

Tumor image analysis using concatenation will be discussed by Noreen (27) using DL. Deep learning models Inception-v3 and DenseNet201, which have already been trained, help this model. Two alternative situations for identifying and classifying brain tumors were investigated using these two principles. After that, the pre-trained model in Inception-v3 was used to extract the properties of numerous Inception modules and concatenate them to categorize brain tumors. The SoftMax classifier was given these characteristics to locate the tumor. A pre-trained DenseNet201 was used to extract properties from various DenseNet Blocks, and it performed well. The SoftMax classifier was then used to combine these attributes to identify the brain tumor. The publicly accessible three-class dataset for brain tumors was used to assess both situations in-depth and objectively. To identify brain cancers, this approach has a precision of 99.34% for Inception-v3 and a positive predictive value of 99.51% for DenseNet201, respectively. For the classification of brain tumors, researchers discovered that feature concatenation leveraging pre-trained models outperformed current deep learning and machine learning approaches.

Razzak (13) highlights the effective brain tumor segmentation with a multiscale two-pathway cluster CNN in neuroimaging. Precision and heftiness of brain tumor sectioning are crucial to diagnosis, therapy planning, and evaluation of treatment outcomes. Automated brain tumor segmentation generally relies on hand-created characteristics. Even in the medical area, accessing labeled data is sometimes challenging for convolutional deep learning algorithms like Deep CNN. A CNN architecture incorporating local and global properties has been developed to segment brain tumors. Equivariance is enforced in the Two-sided CNN architecture to decrease the risk of overfitting. Cascade architecture is included in our two-pathway group CNN by using a rudimentary CNN as an extra source and concatenating it on the final layer. Group CNNs have been shown to improve the overall performance of the BRATS2013 and BRATS2015 datasets models, according to the validation of multiple architectural models.

Using DL approaches, Sultan (28) quantified multi-image categorization. MRI is frequently utilized because of its high-quality images and the fact that it uses no ionizing radiation. As a branch of machine learning, DL has lately shown impressive results, particularly in classification and segmentation issues. A convolutional neural network-based DL model for detecting several forms of brain cancers. It first categorizes the tumor into meningioma, glioma, and pituitary tumor, and later, the Glioma grade (Grade II, Grade III, and Grade IV) is determined.

Transfer learning has been used by Swati (29) to improve brain tumor retrieval from MR images based on content. Using handmade features, we may create an extraction technique for low-level and high-level features that reduces their disparity. When it comes to feature representations, deep learning is an excellent option since it can include the extraction of features into the self-learning procedure. CFML, a deep convolutional neural network (CNN), and a VGG19-based innovative parameter extraction method are utilized to determine whether the query and database images are comparable. Extensive research was done using a publicly available CE-MRI dataset comprising 3,064 images of 3 different forms of brain cancers (namely glioma, meningioma, and pituitary tumor) from 233 individuals. This method requires a minimum preprocessing for the CE-MRI dataset and is resilient in 5-fold cross-validation tests. It can reach a 5-fold mean average accuracy of 0.9654 and beats current CBIR systems by a wide margin. No features are created in the procedure.

Wang (30) explains dynamic medical image segmentation by combining DL with fine-tuning. CNNs were integrated into a segmentation process that employed bounding boxes and scribble-based classification to concentrate on deep learning. Network and interaction-based uncertainty is taken into account while fine-tuning. There are two new applications for this framework, such as the 2-D segmentation of various organs from fetal MR slices with only two annotated versions for testing, as well as the 3-D segmentation of a brain tumor core (excluding edema) and a whole brain tumor (including edema) from two separate MR sequences with only the tumor core annotated for training in one MR sequence each.

A deep learning system that uses transfer learning methods was employed by Rehman (31) to diagnose brain cancers. For example, Meningioma, glioma, and pituitary cancers may be detected using three different CNN designs (Alex Net, Google Net, and VGG Net). MRI slices of the brain tumor dataset are used in each research to explore transfer learning methodologies such as fine-tuning and freezing. Alex Net is tasked with investigating transfer learning mechanisms as part of its first inquiry. If a fine-tuned Alex Net technique is necessary, we have analyzed several criteria to ensure the finest possible execution and the most accurate results. SGDM, Adam, and Rms Prop are three key solutions created for the network with varying cluster sizes and approval frequencies. The Google Net model is utilized in the second review to explore the techniques of exchange learning illustrated. A few assessments use the pre-trained Google Net’s tweaking strategy to try to alter the limits. A remarkable 98.04 percent success rate has been attained. This is the third and last examination of CNN’s VGG16 architecture, in which the effectiveness of the CNN techniques is examined. Using the calibrated VGG15 classifier, the data reveals the accuracy of various companies using different solvers and different restrictions. Depending on VGG16, the best classifier can be found. In this case, 98.69 percent of the facts are distinguishing evidence.

Afshar (27) classified the tumor as a Brain Tumor with a Capsule Network. CNNs cannot handle input alterations very well if you do not have much training data. New architectures for machine learning, known as Caps Nets, have been developed to address the shortcomings of CNNs and revolutionize deep learning systems. In the context of medical imaging datasets, such as brain MRI images, CN is resilient to rotational and axial transformation and needs less training data, as is the case here. The activity vectors of neurons may represent different posture parameters in a capsule, and the longer these activity vectors are, the more likely an object is to exist. Many CNN flaws can be traced back to the level of pooling. A more appropriate criterion, known as routing by consensus, is instead employed in Capsule networks. Although their coupling coefficients vary, this criterion directs outputs to all parent capsules in the subsequent layer. The method effectively resolves CNNs in brain tumor categorization. Table 2 provides a comparative literature evaluation of notable segmentation of human brain tumor features, modalities, datasets, the technology used, and constraints.

**Table 2:** A comparative literature evaluation summarizes brain tumor segmentation methodologies, datasets employed, technologies utilized, and the limitations identified from recent notable studies.

Ref.	Propose model	Characteristics	Modality	Dataset	Used technology	Limitations
Zhou et al. (1)	CNN and MM data aquisition	Expand the connection speed of the network Assuage the problem of overfitting Loss function improved	MRI	MICCAI BraTS (2018).	Multimode state 3D CNNs	2D CNN on the training layer is problematic for adopting the image
Weinberger et al. (2)	Improve the residual network to segment Multi-model	Shorten the distance between information paths and reduce cumulative error during training, increase information diversity	MRI	BRATS2015	Residual DN with middle regulation	DSC Score with ResNet is lower
Sobhaninia et al. (3)	Reduced architecture for multiple interconnection in stacks.	Simple reducing Net (SRNet) is improved with the original UNet The number of parameters is reduced to 4/5 Improve the loss of information with SCN	MRI	BRATS2015	Bridge connection method, Stacked Network, SRNet	Overfitting problem: stacked UNet RUnet uses too many parameters and more computation resources.
Liu et al. (42)	Expand learning dataset with correlation GANs	Generate synthetic MRIs of different modalities Combine the slice-level glioma subtype Classify the glioma molecular subtype	GAN augmented MRIs	TCGA-GBM and TCGA-LGG	GAN augmented MRI as a dataset with a 2D CNN	Signal-to-noise ratio (STNR) autoencoder feature with original MRIs, Accuracy is lower
Diaz-Pinto et al. (4)	Combined deep handcraft parameters	The classification process is improved with SVM as a classifier	MRI	Collect from the Iraqi center for research and magnetic resonance of Al-Kadhimain Medical City.	MGLCM-DF method, convolutional filters, AlexNet, GoogleNet, SqueezeNet	A vast difference occurs in the inaccuracy results with the BRATS 2013 datasets.
Goodenberger and Jenkins (5)	HS-SVM architecture	MRI slices pixel resolution processed at such a slow pace with the increment of iteration. Design an extreme learning machine algorithm	MRI	Not mentioned	Use ELM algorithm use FAHS-SVM	Tumor size affects the accuracy of segmentation
Menze et al. (6)	Paroxysmal epilepsy with C-LSTM	Detect seizures and tumors in the human brain. solve the overfitting problem	MRI	Not mentioned	Deep convolutional LSTM model	Measurement noise and accuracy are not improved
Bakas et al. (7)	Deep ripple autoencoder approach	Accuracy, Specificity, and sensitivity improve. Combining DNN and other autoencoders with the same dataset enhances the performance.	MRI	RIDER	DWA – DNN classifier	Time-consuming process and handling problem.
Myronenko (8)	Concatenation-based approach	The features from Inception are extracted and concatenated with classification DensNet201 is used to extract the features. Classification of different types of tumors	MRI	CE-MRI	DensNet deep learning model, inception model used	It blends the most suitable algorithms, AdaGrad and RMSprop.
Jiang et al. (9)	Multiscale two-pathway cluster CNN	Exploit local features and global contextual features Reduce instabilities and overfitting by sharing	MRI	BRATS2015 BRATS2013	Two-pathway CNN architecture	2PG-CNN gives low accuracy
Isensee et al. (10)	Multiple classification and extraction	Differentiate the three subdivisions of gliomas 100% accuracy achieved with 500 iterations. achieved lower loss (better)	MRI	Collected from Nan Fang Hospital and General Hospital, Tianjin Medical University	Custom deep neural network, and two dropout layers used	Grade III glioma grade gives 95% accuracy, while other grades give 100% accuracy
Isensee et al. (11)	Retrieval using evidenced learning	Enhance retrieval performance by using a fine-tuning strategy. Require minimal preprocessing	MRI	CE-MRI	Deep CNN with VGG19-based parameter extraction construction	The tumor is roughly outlined
Oktay et al. (12)	Image specific fine tuning	Robust model Weighted loss function improves Memory Efficient Short influence time	MRI	BRATS	DL-based interactive segmentation framework, scribble-based segmentation	Noise ratio and feature sharing, overfitting problem with High Res3DNet, deep medic gives lower performance
Zhou et al. (14)	Automated classification With DL	In-depth, perilous factors distressing the fine-tune approach Enhanced performance on image classification Beat the out-of-date ML models	MRI	ImageNet	Alex Net, Google Net, VGG Net	AlexNet and GoogleNet give lower accuracy
Hatamizadeh et al. (15)	Energized algorithms	Perfect fitting Reduce the number of parameters Reusability of weights Increase the efficiency and effectiveness of the image It creates more or supplementary data where limited data is provided	MRI	Not mentioned	2D CNN	The SVM classifier gives lower accuracy than expected

Tumor segmentation in medical imaging is fundamental for computer-assisted diagnosis and treatment planning. In the past few years, a significant amount of work has been focused on optimizing neural architectures for this task, with Res-UNET variants and deep supervision strategies leading to substantial improvements (10, 32–41). The work presented in (17), which spans 2022 to 2025, with enhanced Res-UNET frameworks, shows a significant advancement of tumor segmentation regarding accuracy, robustness, and computational efficiency. The Res-UNET architecture is an extended version of the classic U-Net architecture. Still, it adds residual connections, which allow gradients to propagate better during backpropagation and alleviate the vanishing gradient problem, allowing deeper networks to be trained. Residual connections are particularly advantageous in medical image segmentation tasks, as fine-grained structural details must be captured at all scales. Sahayam et al. (36) residual built a dual attention mechanism with deep supervision into hybrid multi-resolution U-Net models. They trained their model on brain tumor segmentation with MR images and substantially improved Dice Similarity Coefficient (DSC) and Intersection over Union (IoU) metrics. Residual and attention modules allowed for more fine-grained localization around tumor edges, and deep supervision facilitated fast convergence during training.

Wang et al. (37) performed liver segmentation and recently introduced a Multi-scale Attention and Deep Supervision-based 3D UNet (MAD-UNet). Their architecture simultaneously improved spatial and contextual representation by replacing pooling layers with convolutional operations and adding long-short skip connections. Their model was evaluated on datasets such as LiTS17 and 3DIRCADb, with DSC scores above 0.97, indicating a higher score than baseline U-Net and standard Res-UNET models. Deep supervision is adding auxiliary losses to an intermediate layer of a network. This can contribute functional gradients at each level of the encoder-decoder pathway, avoiding any instability in training and ensuring the hierarchical features can be learned. This is especially helpful to segment complex or small tumor regions that risk being overlooked in the final layer-only supervision.

Ma et al. (38) designed an Attention R2U-Net-based Multi-Task Deep Supervision (MTDS) framework for brain tumor subregion segmentation. Their model outperformed regional accuracy and refined the boundaries by incorporating auxiliary losses at different decoder levels corresponding to each segmentation task (i.e., edema, necrotic core, enhancing tumor). The attention helped achieve higher feature discrimination between different tumor types. Cui et al. (39) adopted a similar philosophy, which presented Encoding Feature Supervised UNet++. They introduced supervision signals for each encoder stage that encouraged several epochs of informative feature extraction at the start of the network. On liver tumor datasets, ES-UNet++ outperformed UNet++ by 5.7% in the Dice score. These findings reveal why deep supervision not only increases the accuracy of our models but also helps in generalizability across datasets with different imaging phenotypes.

Recent work has improved performance using attention modules, multi-scale processing strategies, and Res-UNET. This design incorporates attention mechanisms like channel and spatial attention blocks to encourage the network to pay attention to relevant tumor regions while ignoring the surrounding background. This is crucial in medical imaging, where tumors are usually of low contrast. Huang et al. (40) evaluated the brain tumor segmentation datasets with different architectures such as UNet, Res-UNET, Attention Res-UNET, and UNet. They found that integrating Res-UNET and attention mechanisms led to a marked improvement in performance compared to traditional architectures, especially concerning discriminating between overlapping tumor subregions. The attention significantly improved boundary detection and decreased false positives.

Another innovation in DeepLab is the Atrous Spatial Pyramid Pooling (ASPP), which helps obtain feature representations at multiple scales and captures context. It has been incorporated into Res-UNET architectures to manage tumors with irregular shapes and sizes effectively. Although ASPP has been more commonly deployed in segmentation tasks outside tumors (e.g., lung, pancreas), it is beginning to be used in oncology, condensing local and global contextual features. Many of these streamlined architectures have been tested on public datasets (e.g., BraTS [brain tumors], LiTS [liver tumors], and ISLES [stroke lesions]). BraTS has become the canonical benchmark for evaluating the performance of glioma segmentation algorithms. In various challenges, the hybrid schema of Res-UNet with deep supervision and attention modules consistently yields the best solutions. For example, Behzadpour et al. (41) proposed a model built upon an EfficientNet encoder within a Res-UNET architecture with additional deep supervision and attention blocks. On BraTS2021, their approach achieved a whole tumor Dice score of 0.91 and a tumor core score of 0.85. These results highlight the importance of encoder choice, intermediate supervision, and context-aware modeling in achieving clinically relevant segmentation performance.

## 4 Proposed methodology

**Figure 4:**
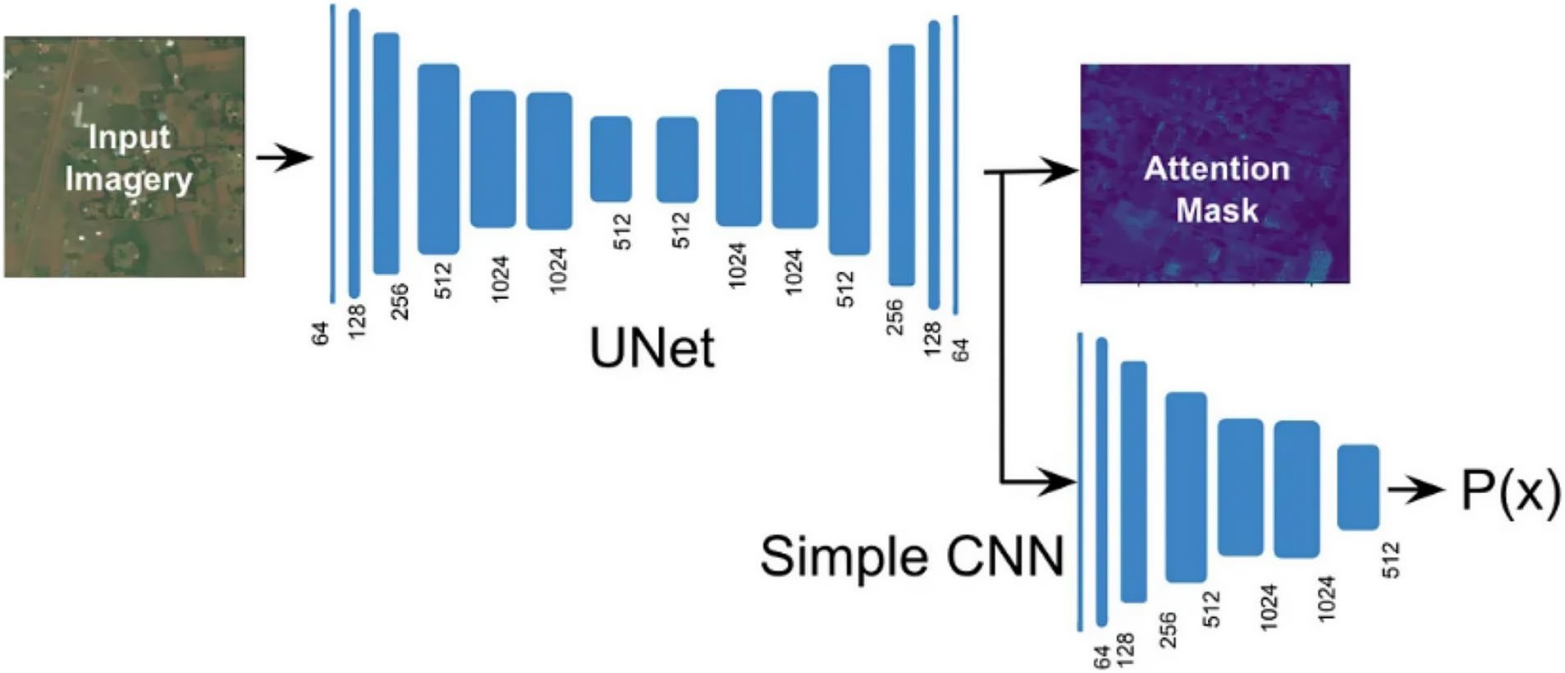
Demonstration of a conventional U-Net model combined with CNN layers and an attention mask for emphasizing important features to improve segmentation precision during training and inference phases.

For this study, we exploited a publicly available dataset (32). In the recent literature, we have found several improvements to the original U-Net architecture to try to build upon its basic strengths. These are introducing attention mechanisms, residual connections, and multi-scale feature integration. We further evolve our traditional U-Net with residual blocks to improve gradient propagation and extract more levels of features. Additionally, we employ deep supervision to enforce learning at intermediate decoder layers. The presented evolution of U-Net, leading to our proposed Res-UNET with deep supervision, gives a systematic approach to improving accuracy while remaining computationally efficient in training encounters. U-Net has been analyzed to find the most effective NN design. Each model is described briefly in the sections that follow. The U-Net design (Figure 4) separates the encoder and decoder by a symmetric U shape. Initially, the input space is transformed into a smaller space by the shrinking route (encoder). The encoder’s modular structure is made up of repeated convolution blocks. The transformations are divided into two smaller blocks. A convolutional layer with features 3 × 3 × 3 and a cadence of 2 × 2 × 2 is used to reduce the feature space of the input matrix by a factor of two, then occurrence normalizations and Leaky ReLU activation with a negative slope of 0.01 are used (dark blue block). A similar set of operations is used to modify the following subset of features, but the convolutional layer has a stride of 1 × 1 × 1 (light blue). The decoder begins after the featured graph’s spatial dimensions are reduced to 2 × 2 × 2.

However, the decoder aims to improve the spatial dimensions by lowering the encoder feature space. Three smaller blocks combine to form the decoder block. There are two methods for extending the feature map’s spatial dimensions. The first uses an inverted convolution with kernels and strides of 2 × 2 × 2 and 2 × 2 × 2. A convolutional layer with filter size 3 × 3 × 3 and a cadence of 1 × 1 × 1 is used to convert the up-sampled feature map, which is then combined with the encoder feature space from the same spatial level. Then, instances of normalizations and Leaky ReLU activation with a negative slope of 0.01 are used (light blue) (32). In contrast, deep supervision can be employed to compute loss functions for outcomes from lower decoding levels.

**Figure 5:**
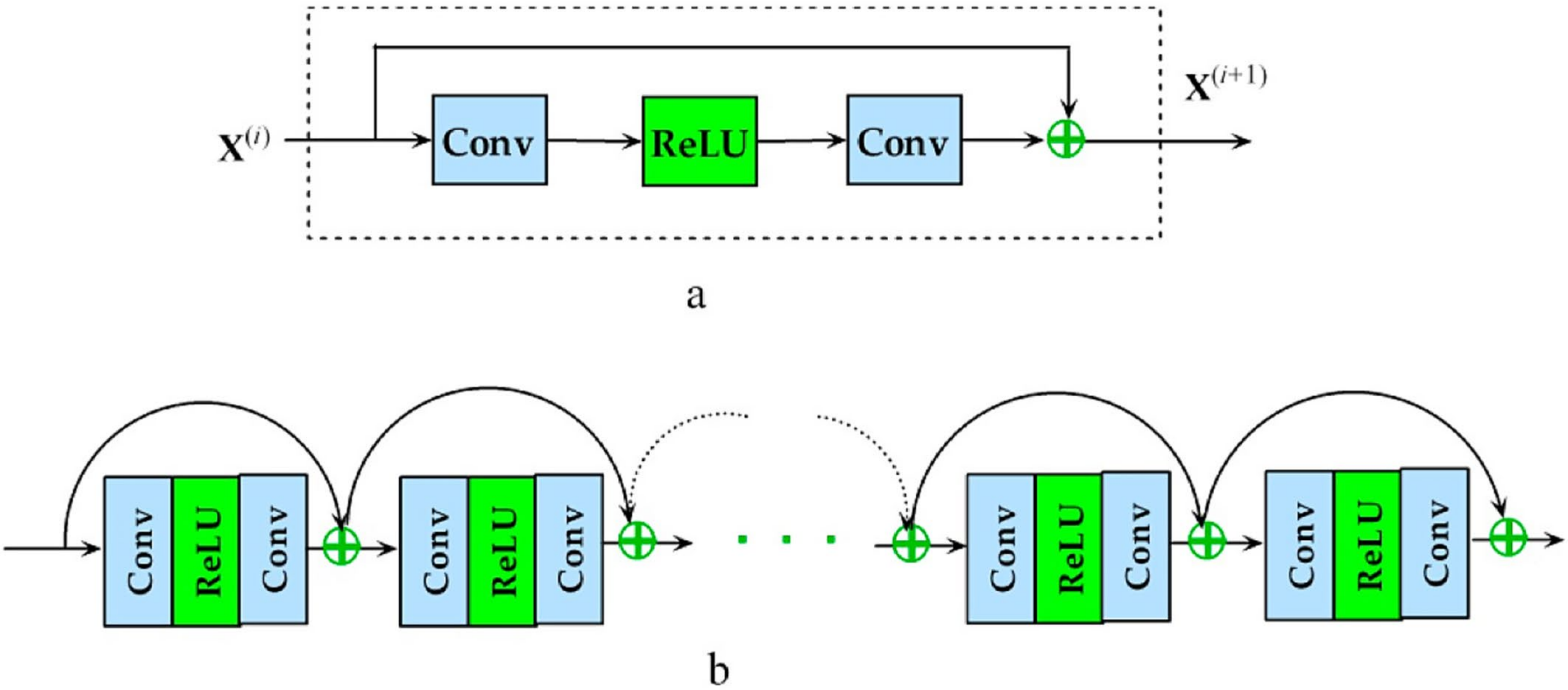
Representation of the proposed deep Residual U-Net autoencoder architecture utilized in experiments, highlighting encoder-decoder structures with autoencoder regularization, employing residual connections to enhance gradient flow and improve training efficiency.

BraTS 2018 contest winner Residual Autoencoder U-Net is an AS-U-Net with autoencoder regularization that re-engineers the encoder frames and attaches a VAE subsidiary in the demultiplexer that recreates the input and has a regularization impact (see Figure 5). There are two convolutions with class normalization and activation of the ReLU in each frame, accompanied by an additional identity backpropagation. The decoder has a striking resemblance to the encoder, but each spatial layer only has one unit. Before adding an encoder extracted features from a higher spatial layer, each decoder block reduces the number of bands by a factor of 2 and doubles the spatial dimension. A low-dimensional vector of 256 is used in the decoder’s VAE section to decrease the congestion of extracted features. Samples from the Gaussian distribution with specified mean and standard deviation are then selected from this distribution to rebuild input image dimensions, similarly to decoder construction.

**Figure 6:**
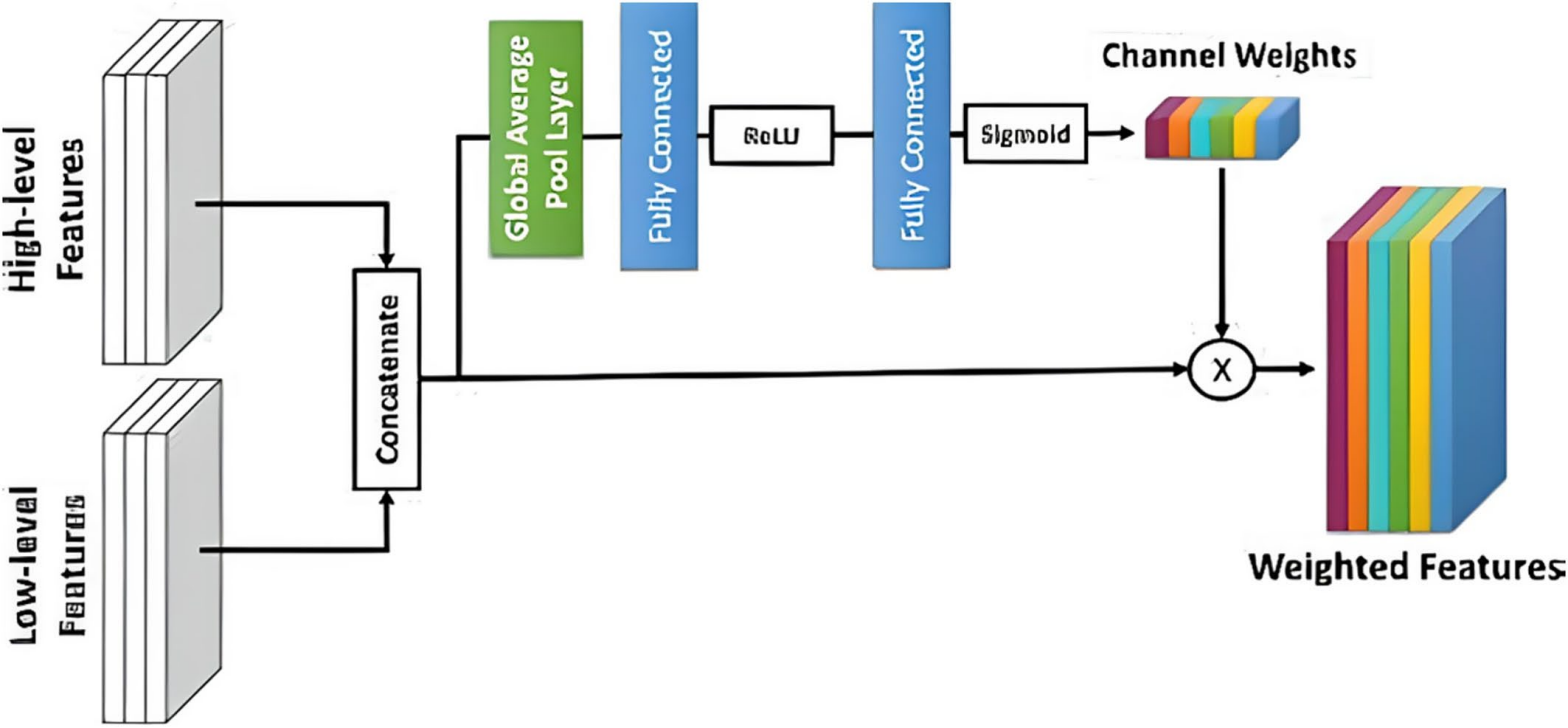
Illustration of an enhanced attention unit utilized in the experiments, emphasizing the weighted selection of relevant features in the decoder block of attention U-Net to improve segmentation accuracy.

In UNETR (see Figure 6), three-dimensional CN is replaced by attention, which postpones ViT to three-dimensional convolution layers. An input area is partitioned into a series of non-overlapping chunks and placed onto a subspace (with 768 parameters) using a sequential layer, and the positional embedding is provided. A multi-head self-attention encoder then processes this data.

The decoder portion of the Attention U-Net adds an attention gate to the basic U-Net, increasing its functionality. Before concatenating in the decoder phase, the concentration gate transforms the subset of features from the encoder. It uses the environment of the preceding decoder block’s feature map to understand which portions of the encoder feature space are most significant. The encoder feature map is multiplied by the attention gate’s parameters to accomplish this. Put another way, the NN weights fall between 0 and 1, indicating how much attention it gives every pixel. Motivated by the Reset paradigm in which residual interconnections were presented, a Res-UNet was created. Because of improved gradient flux, residual links assist in training a DNN. There is just one distinction between the basic U-Net and the R-U-Net. Our trials have shown that a simple U-Net produces the most significant outcomes. Next, the encoder depth and convolution effective parameters were optimized. There were six convolution channels at each encoder level, and the depths of the NN U-Net model’s default U-Net structure were utilized as a benchmark. According to our research, raising the encoder’s level to seven and changing the frequency band to 96 to 512 further enhances the baseline rating.

TC represents classes 1 to 4; expanding tumor (ET) represents class 4, and WT represents classes 1 to 2. These three sections partly overlap. Rather than relying on the labeling, the challenge scoreboard depends on the corresponding points. For this reason, we constructed the output feature vector to include three channels (one for each class), which are then converted using sigmoid activation to create the prediction error. The Dice loss was used to optimize each area using a sum of binary cross-entropy or Focal loss. Rather than summing the Dice loss across each sample individually, we utilized the batch form of Dice loss to calculate the loss across all items in the group. Deep supervision is a method that uses several decoder levels to compute the loss function and improve gradient flow. The green bars depict the insertion of two new output units in this study. Labels were quantized using the nearest neighbor approximation to match the spatial morphologies of extra outputs before calculating the more profound supervision loss. In this case, the final loss factor is determined as follows for labeling Yi and forecasts pi for A = 1 to 3, where A = 1 represents the final projection header, A = 2 shows the output head on the subsequent decoding level, and A = 3 represents the output head well before preliminary.

## 5 Experimental results and discussion

Throughout inference, the input quantity may be of any size rather than the predetermined patch size of 128 × 128, as during the training stage. Because of this, we employed a feature extraction inference 2 with a training patch as the frame and consecutive frames overlapped by exactly half of a patch. Consequently, as in the initial NN-U-Net study, the values of the center voxels are given a larger relevance than the weights of the overlapping areas when averaging the predictions. A segmentation-specific loss function arises from the Dice Similarity Coefficient (DSC), which assesses the overlap between predicted and ground truth segmentation masks. Incorporating a combination of class weights made the most impactful performance improvement. This is particularly beneficial in medical image segmentation since there is often class imbalance, and it weighs the importance of smaller, pertinent areas (like tumors) in prediction. Deep Supervision is achieved by adding other loss functions to intermediate layers of the network. Guided back-propagation passes the gradient back through all layers to provide hints of a CNN spread over most levels instead of merely relying on guidance from the output layer. These strategies benefit deep architectures like Res-UNET, which suffer severely from vanishing gradients and need robust multi-scale learning. One recognized strategy to increase the reliability of forecasts is to improve the time spent in the test environment. Since there are eight different x, y, and z axis combinations, we produced eight variants of the input volume during inferences. To turn the predictions back to the original input volume orientation, we execute inference for each iteration of the input volume and utilize flips on forecasts used for the input volume. Finally, we calculated the average of all forecasts’ probabilities. It returned the three interrelated areas to their respective classes after optimizing them. Classes are returned to their original state by using the following techniques: If the WT likelihood is less than 0.45, the voxel is assigned the category of “background.” Alternatively, if the probability of TC is less than 0.40, the voxel is assigned the category of ED. Finally, if the probability of ET is less than 0.45, it is assigned the category of 1 or NCR, respectively (ET). The accompanying post-processing approach was also used: For constituents with a mean likelihood of fewer than 0.9, change their category to NCR. Next, change all ET voxels into NCR if there are generally just under 73 voxels with those, and their mean likelihood is lower than 0.9. The edge scenario where the prediction of a few voxels with enhanced tumors was avoided using such post-processing, but there were none in the underlying data. As a result of this post-processing, the Dice value for a zero high false forecast was 1, as well as a 0 otherwise, which was helpful to the total result. A systematic hyperparameter tuning process was undertaken to optimize the performance of the proposed Res-UNET model.  We first performed a grid search over various learning rates, weight decay values, and loss function weights. We explored learning rates in the range of 1e-2 to 1e-5 and found the best performance using 1e-4 with the Adam optimiser. Weight decay is a kind of regularization, and we tested weight decay values between 0 and 1e-4 before selecting 1e-5 to balance the overfitting trade-off while keeping convergence stable. Segmentation loss was calculated as a composite of Dice Loss and Cross-Entropy Loss, with the weighting ratio for Dice Loss (*α*) sweeping between 0.5 and 0.8, where the optimal setting of α = 0.7 produced the best trade-off between region-wise segmentation correctness and boundary sharpness.

Hyperparameter tuning for batch and input patch sizes improved memory utilization and contextual representation. Without exhausting the memory in the GPU, a batch size of 2 and a 3D patch size of 128 × 128 × 128 provided the most stable training. Furthermore, early stopping with a patience of 10 epochs was employed to prevent overfitting, and a ReduceLROnPlateau learning rate scheduler was applied to decrease the learning rate if the validation loss showed no improvement. The final hyperparameters were validated on a hold-out set of 20% of the training data, and 3-fold cross-validation was utilized to assess the extent of generalizability and stability of the chosen configuration.

**Figure 7:**
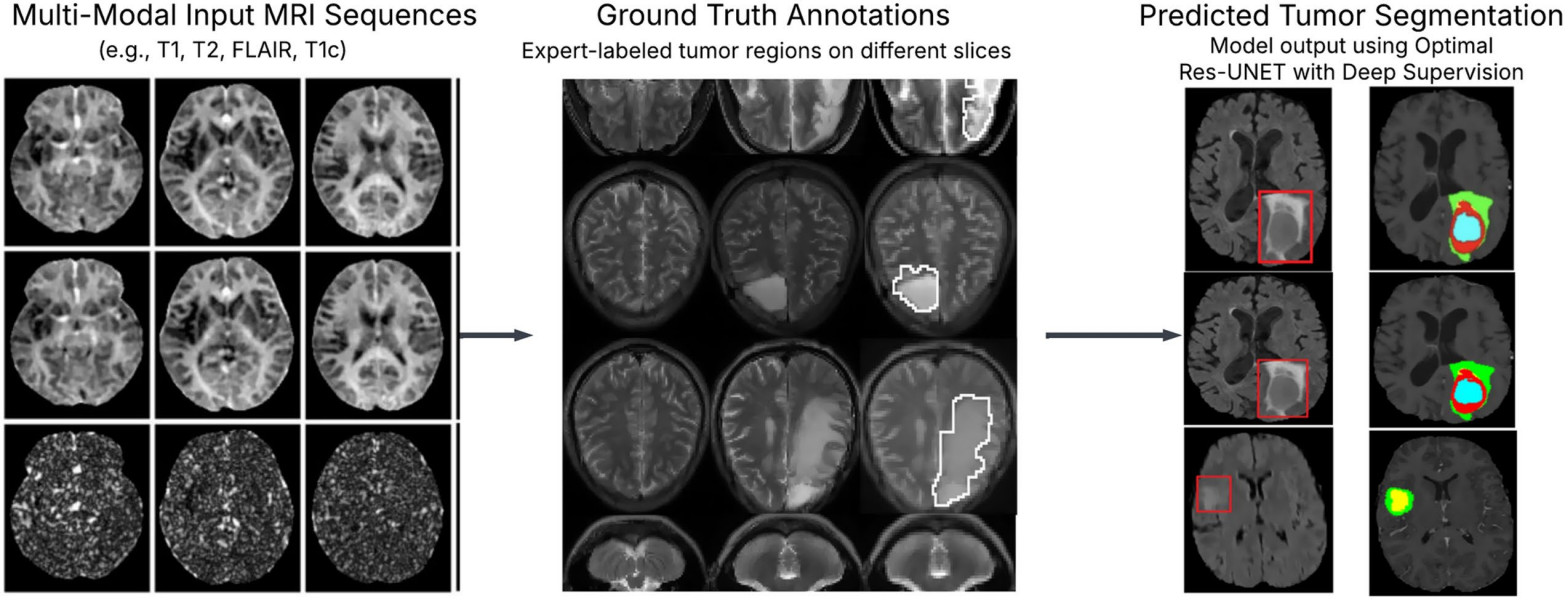
Experimental results of brain tumor segmentation: tumor segmentation results using optimal Res-UNET with deep supervision show high alignment with ground truth. Figure 1 L1, L2, L3 metrics and total loss visualization over epochs.

Our experiments with three different U-Net versions led us to use the better method for input rebuilding: foundation U-Net, UNETR, and U-Net plus autoencoder normalizations, which all use the same U-Net structure but use a ViT interpretation for 3D convolution operation instead of U-Net. Basic U-Net received the highest, according to the findings. Residual Autoencoder U-Net’s learning duration is 3 times higher than that of U-Net, even though the rating is almost the same. As a result, we chose to focus our research on the U-Net structure. Several U-Net design adjustments, including decoder attentiveness, deep supervision, residual links, and a drop clog, were evaluated in the following experiments. The updated loss function has also been tested using Focal loss rather than cross-entropy, such that Focal loss with Dice is the error term. The deep supervision was the sole addition to the U-Net 91%, which improved the 5-fold average Dice score by 91%, according to the data provided. Table 3 shows comparative results between the implemented Conventional U-Net and Residual Autoencoder U-Net, detailing Dice scores across various cross-validation folds. Figure 7 depicts the experimental outcomes of brain tumor segmentation, visually demonstrating performance differences across various tested U-Net architectures.

**Table 3:** A comparative performance analysis between the implemented Conventional U-Net and Residual Autoencoder U-Net, detailing Dice scores across various cross-validation folds, indicating that conventional U-Net generally achieved higher Dice scores, outperforming Residual Autoencoder U-Net.

Experiment model implementation	Conventional U-Net	Residual Autoencoder U-Net
Fold 0	0.9297	0.8977
Fold 1	0.9698	0.8999
Fold 2	0.9189	0.9092
Fold 3	0.9387	0.9398
Fold 4	0.9456	0.9433

Lastly, we evaluated the encoder modifications for the U-Net with deep oversight. It adopts the architectural rationale from the NN U-Net platform, where the network depth is six and the number of convolution units at every encoder level is 32 to 320. We used a depth seven encoder, expanded the number of layers from 64 to 512, and used a one-hot encoder for salient voxels to validate the input space-Net with deep supervising 91% has a slightly better rating if each one of the adjustments is applied individually, but if all adjustments are used together and the score improves further 91%. A post-processing method was also tested. Table 4 illustrates epoch-wise validation losses for different layers (L1, L2, L3) and total loss across training epochs. To make sure the clinical or real-time use of the proposed Res-UNET with deep supervision is usable, we assessed the computational performance. We trained the model on an NVIDIA RTX 3090 GPU with a batch size of 2, with convergence around 75 epochs. The average inference time for each volume was 1.8 s. The introduction of deep supervision and residual connections added extra complexity. At the same time, reviews demonstrated that the model achieved efficient memory usage and a manageable number of parameters, thus allowing for scale across high-resolution MRI scans. Additionally, we include deep supervision from intermediate decoder stages to improve our proposed architecture’s learning power and convergence property. This method uses auxiliary loss functions on specific layers, incentivizing early feature maps to contribute meaningfully to the final prediction. In theory, deep supervision enhances gradient flow in deep networks by supplying direct supervision signals to preceding layers, helping to alleviate the vanishing gradient issue and promoting faster and more stable training convergence. The performance of deep supervised models based on empirical results shows lower training loss and better segmentations in all the different tissue types/tumor subregions. These advantages can be seen in a comparative visualization of loss convergence with and without deep supervision. Apart from architectural enhancements, we also utilize targeted post-processing techniques to improve segmentation robustness. In particular, connected component analysis is used to discard the small isolated false-positive spots that could occur in non-tumoral regions. Moreover, morphological actions like dilation and erosion improve tumor borders and maintain small or divided tumor predictions. This can significantly enhance the detection of smaller tumor regions and diminish false positive rates, leading to much neater and clinically consistent segmentation outcomes. These design choices collectively allow us to build a more reliable segmentation framework closer to being suitable for deployment in high-stakes medical imaging settings.

**Table 4:** Epoch-wise validation losses for different layers (L1, L2, L3) and total loss across training epochs, showing continuous improvement and validation accuracy incrementally through training epochs.

	L1	L2	L3	Total loss
Epoch 0	72.81	72.71	85.23	0.9542
Epoch 5	74.22	76.79	83.33	0.7793
Epoch 10	84.49	80.92	89.63	0.5207
Epoch 15	87.2	80.88	90.63	0.4771
Epoch 18	86.58	83.46	89.31	0.4728
Epoch 22	88.7	84.11	91.81	0.4033
Epoch 26	89.98	85.97	91.22	0.3924
Epoch 28	90.79	87.35	95.06	0.3694
Epoch 29	91.69	89.5	96.88	0.3132

In past BraTS versions, it has been shown that eliminating tiny areas of increased tumor may improve the ultimate result. So, since the Dice score for predicting zero false positives is one if there is no enhancing tumor present in the label, and zero if there is. After performing five-fold cross-validation, we discovered that the best approach was to look for ET-connected components, then swap out smaller than sixteen voxel elements with a mean likelihood lower than 0.9 for NCR-class parts, and finally, swap out all ET-class voxels with a probability lower than 0.9 for NCR. Table 5 summarizes cross-validation results incorporating deep supervision and encoder optimizations.

**Table 5:** Cross-validation results incorporating deep supervision and encoder optimizations, revealing the proposed model’s superiority in segmentation performance compared to state-of-the-art approaches, with a consistent Dice score averaging above 91%.

Experiment model implementation	Deep supervision	Deeper UNET	Channels results	Mean
Fold 0	0.9251	0.9217	0.9227	0.9089
Fold 1	0.9365	0.9270	0.9335	
Fold 2	0.9435	0.9343	0.9443	
Fold 3	0.9698	0.9635	0.9575	
Fold 4	0.9494	0.9135	0.9672	

## 6 Discussion

This study aimed to present an improved brain tumor segmentation framework, which has been built upon an Optimal Res-UNET-based architecture combined with deep supervision, particularly for effective and robust brain tumor segmentation during multi-modal MRI. The architecture employs residual learning, multi-scale feature fusion, and auxiliary loss supervision for effective feature representation, improved gradient flow, and efficient segmentation performance. Residual connections mitigate one of the fundamental problems with deep networks: vanishing gradients. Residual blocks help gradients to propagate through networks more easily via shortcut connections, with activated identity mappings. In contrast, gradients are backpropagated (32)—supporting the training of deeper models without performance degradation. This modification adds to structural stability, requiring only a few fine-tunings while retaining semantical information and low-level features important in medical imaging.

Deep supervision (e.g., auxiliary losses applied at intermediate decoder stages) is an essential architectural innovation. For example, deep supervision reduces overfitting and enhances generalization by propagating error signals to several network depths, motivating the first layers to learn discriminative visual features (32). The introduction of deep supervision led to significantly faster convergence and better DSC values across the tumor subregions, with particular improvement for tumor core detection, where the irregular shape and small size can lead to false negatives during detection. The metric we used to assess segmentation performance is known as Dice Loss. This differentiable metric is based on the Dice Similarity Coefficient, a measure of the overlap between the predicted and the true mask. Dice loss is more suited for tasks with a high-class imbalance in medical segmentation as it supports high weight on correct smaller structure segmentation (34). In addition, we used post-process techniques to further clean up the segmentation results. Through connected component analysis, small disconnected false positives were eliminated, and morphological operations (dilate and erode) reduced spatial incoherence in predicted masks. These were important to remove noise and increase the identification of small tumor areas, which are usually missed in coarse segmentations.

Traditional U-Net offers a solid yet straightforward baseline for biomedical image segmentation, but performance-increasing architectures have been presented. Example configurations have learned to work well on a given dataset, as in the case of nnU-Net, which is set out of the box and can be trained without user-defined outcomes (10). In contrast, TransUNet integrates transformer-based encoders into the U-Net to capture long-range dependencies, resulting in better contextual reasoning (35). Similarly, the Attention U-Net uses attention to guide the network toward relevant regions of interest. Compared to these, our model achieves a good trade-off between architectural sophistication and performance using residual learning and deep supervision. The proposed solution is the Optimal Res-UNET with Deep supervision; it successfully tackles the abovementioned problems in medical image segmentation. These architectural improvements enhance the learning dynamics of the model, sharpen boundary segmentation, and improve the accuracy of heterogeneous tumor subregion segmentation. Moving forward, we hope to validate the generalizability of our model across institutions and Supplements to other organ-specific segmentation tasks.

## 7 Conclusion

In this work, we have overcome the limitations of traditional UNet-based architectures for tumor segmentation through our optimal Res-UNET integrated with deep supervision. We enhance segmentation by utilizing residual connections for floating gradients within the decoder and introducing a series of auxiliary loss functions throughout the sequential decoder. This deep supervision approach alleviates the vanishing gradient issue, boosts the convergence rate, and better delineates tumor boundaries more clearly in heterogeneous and low-contrast areas. We conducted a thorough literature review, particularly focusing on recent developments after 2017, including nnU-Net, Attention U-Net, and TransUNet, which employ novel approaches such as dynamic configuration, attention mechanisms, and transformer-based feature extraction. Built on these architectures, our approach was compared to others and demonstrated that integrating residual learning with deep supervision and post-processing operations, such as connected component analysis and morphological refinement, significantly enhanced the model’s robustness and sensitivity to small tumor regions. Moreover, experimental results show that the proposed method achieves higher Dice accuracy and boundary precision than conventional UNet and its variants. More expressive feature learning via enriched input representation, increased convolutional channels, and structured post-processing to reduce false positives improved the overall F1 score. By integrating deep supervision, residual encoding, and tailored post-processing, this work illustrates the positive impact of these elements on brain tumor segmentation. It offers a more robust solution with achievable scalability. We anticipate that our future work, including designing more sophisticated politeness models, will validate its performance across multi-institutional and other multi-domain datasets and demonstrate the effectiveness of techniques like domain adaptation and advanced architectures such as hybrid CNN-transformer models. A greater collaboration between machine learning and computer vision researchers will also be essential to bridge the gap between these advancements and the establishment of clinically robust systems. Deep Learning principles were introduced utilizing UNET-based models and similar methodologies. This research concentrated on the key challenges neural network-based UNETs face in brain tumor segmentation and future strategies for overcoming those challenges. Subsequently, in the study section, we provided a comprehensive review of existing literature, emphasizing works published post-2017. Another distinction of this article from other papers is its use of Deep Learning-based UNET to identify and classify brain tumors from a Computer Vision/Machine Learning perspective. From this standpoint, we highlight the root causes of the difficulties encountered in this approach and propose effective future directions based on insights from diverse scientific fields. We have examined and advocated several solutions to analogous issues in related scientific domains. Finally, different U-Net variations have been tested, including the standard U-Net and Attention U-Net, along with various architectural modifications and training scheduling adjustments, such as deep supervision and Focal loss. Combining U-Net with deep supervision and an additional salient input channel encoded using one-hot decoding, an increased number of convoluted channels, and an appropriate post-processing technique is the most effective method to achieve even better results. We conclude that brain tumor segmentation can significantly benefit from NN-based UNET through increased collaboration with Computer Vision and ML research groups.

## Data Availability

Publicly available datasets were analyzed in this study. This data can be found here: https://figshare.com/articles/dataset/brain_tumor_dataset/1512427.

## References

[1] Zhou C Ding C Wang X Lu Z Tao D. One-pass multi-task networks with cross-task guided attention for brain tumor segmentation. IEEE Transactions on Image Processing. (2020) 29:4516–29. doi: 10.1109/TIP.2020.2973510 32086210

[2] Weinberger P. Yosifov M. Fröhler B. Kastner J. Heinzl C., “The long journey to the training of a deep neural network for segmenting pores and fibers,” 11th Conference on Industrial Computed Tomography (iCT) 2022, 8-11 Feb, Wels, Austria. e-Journal of Nondestructive Testing. 27. doi: 10.58286/26610

[3] Sobhaninia Z Rezaei S Karimi N Emami A Samavi S. Brain tumor segmentation by cascaded deep neural networks using multiple image scales. 2020 28th Iranian Conference on Electrical Engineering (ICEE). Tabriz, Iran. (2020). p. 1–4.

[4] Diaz-Pinto A Alle S Nath V Tang Y Ihsani A Asad M., “MONAI label: a framework for AI-assisted interactive labeling of 3D medical images,” Medical Image Analysis (2024) 95:103207. doi: 10.1016/j.media.2024.103207 38776843

[5] Goodenberger ML Jenkins RB. Genetics of adult glioma. Cancer Genet. (2012) 205:613–21. doi: 10.1016/j.cancergen.2012.10.009, PMID: 23238284

[6] Menze BH Jakab A Bauer S Kalpathy-Cramer J Farahani K Kirby J. The multimodal brain tumor image segmentation benchmark (BRATS). IEEE Trans Med Imaging. (2015) 34:1993–2024. doi: 10.1109/TMI.2014.2377694, PMID: 25494501 PMC4833122

[7] Bakas S Akbari H Sotiras A Bilello M Rozycki M Kirby JS. Advancing the Cancer genome atlas glioma MRI collections with expert segmentation labels and radiomic features. Scientific Data. (2017) 4:170117. doi: 10.1038/sdata.2017.117, PMID: 28872634 PMC5685212

[8] Myronenko A., “3D MRI brain tumor segmentation using autoencoder regularization, In: Andriy M Alessandro C Spyridon B Hugo K Farahani K Mauricio R. Brainlesion: Glioma, Multiple Sclerosis, Stroke and Traumatic Brain Injuries. Cham: Springer International Publishing. (2019). p. 311–20.

[9] Jiang Z Ding C Liu M Tao D. Two-stage cascaded U-Net: first place solution to brats challenge 2019 segmentation task. In: Alessandro C Spyridon B, eds. Brainlesion: Glioma, Multiple Sclerosis, Stroke and Traumatic Brain Injuries. Cham: Springer International Publishing (2020). p. 231–41.

[10] Isensee F Jaeger PF Kohl SAA Petersen J Maier-Hein KH. nnU-Net: a self-configuring method for deep learning-based biomedical image segmentation. Nat Methods. (2021) 18:203–11. doi: 10.1038/s41592-020-01008-z, PMID: 33288961

[11] Isensee F. Jaeger P. F. Full P. M. Vollmuth P. Maier-Hein K. H., “nnU-Net for brain tumor segmentation,” In: Crimi A Bakas S, eds. Brainlesion: Glioma, Multiple Sclerosis, Stroke and Traumatic Brain Injuries. Cham: Springer International Publishing (2021). p. 118–132.

[12] Oktay O Jo S Loic LF Lee M Heinrich M Misawa K., 1st Conference on Medical Imaging with Deep Learning (MIDL 2018), Amsterdam, The Netherlands (2018). Available online at: https://openreview.net/forum?id=Skft7cijM

[13] Imran Razzak M. Imran M. Xu G., “Efficient brain tumor segmentation with multiscale two-pathway-group conventional neural networks,” IEEE Journal of Biomedical and Health Informatics. (2019) 23:1911–9. doi: 10.1109/JBHI.2018.2874033 30295634

[14] Zhou Z Rahman Siddiquee MM Tajbakhsh N Liang J. “Unet++: a nested U-Net architecture for medical image segmentation,” in lecture notes in computer science (including subseries lecture notes in artificial intelligence and lecture notes in bioinformatics) (2018) 11045, 3:–11. doi: 10.1007/978-3-030-00889-5_1, PMC7329239 32613207

[15] Ali H Yucheng T Vishwesh N Dong Y Andriy M Bennett L., “UNETR: Transformers for 3D Medical Image Segmentation.” 2022 IEEE/CVF Winter Conference on Applications of Computer Vision (WACV). Waikoloa, HI, USA: IEEE. (2022). p. 1748–58.

[16] Dosovitskiy A Beyer L Kolesnikov A Weissenborn D Zhai X Unterthiner T., “An image is worth 16x16 words: transformers for image recognition at scale,” International Conference on Learning Representations. Vienna, Austria (2021). Available at: https://openreview.net/forum?id=YicbFdNTTy

[17] Dorent R Kujawa A Ivory M Bakas S Rieke N Joutard S. “CrossMoDA 2021 challenge: benchmark of cross-modality domain adaptation techniques for vestibular schwannoma and cochlea segmentation. Medical Image Analysis. (2023) 83:102628. doi: 10.1016/j.media.2022.102628 36283200 PMC10186181

[18] Futrega M. Milesi A. Marcinkiewicz M. Ribalta P., “Optimized U-net for brain tumor segmentation,” In: Crimi A, Bakas S, eds. Brainlesion: Glioma, Multiple Sclerosis, Stroke and Traumatic Brain Injuries. Cham: Springer International Publishing (2022) p. 15–29.

[19] Li M Kuang L Xu S Sha Z. Brain tumor detection based on multimodal information fusion and convolutional neural network. IEEE Access. (2019) 7:180134–46. doi: 10.1109/ACCESS.2019.2958370

[20] Ding Y Li C Yang Q Qin Z Qin Z. How to improve the deep residual network to segment multi-modal brain tumor images. IEEE Access. (2019) 7:152821–31. doi: 10.1109/ACCESS.2019.2948120

[21] Ding Y Chen F Zhao Y Wu Z Zhang C Wu D. A stacked multi-connection simple reducing net for brain tumor segmentation. IEEE Access. (2019) 7:104011–24. doi: 10.1109/ACCESS.2019.2926448

[22] Ge C Gu IYH Jakola AS Yang J. Enlarged training dataset by pairwise GANs for molecular-based brain tumor classification. IEEE Access. (2020) 8:22560–70. doi: 10.1109/ACCESS.2020.2969805

[23] Hasan AM Jalab HA Meziane F Kahtan H Al-Ahmad AS. Combining deep and handcrafted image features for MRI brain scan classification. IEEE Access. (2019) 7:79959–67. doi: 10.1109/ACCESS.2019.2922691

[24] Jia Z Chen D. Brain tumor identification and classification of MRI images using deep learning techniques. IEEE Access. (2020):1. doi: 10.1109/access.2020.3016319

[25] Liu Y Huang YX Zhang X Qi W Guo J Hu Y. Deep C-LSTM neural network for epileptic seizure and tumor detection using high-dimension EEG signals. IEEE Access. (2020) 8:37495–504. doi: 10.1109/ACCESS.2020.2976156

[26] Kumar Mallick P Ryu SH Satapathy SK Mishra S Nguyen GN Tiwari P. Brain MRI image classification for Cancer detection using deep wavelet autoencoder-based deep neural network. IEEE Access. (2019) 7:46278–87. doi: 10.1109/ACCESS.2019.2902252

[27] Noreen N Palaniappan S Qayyum A Ahmad I Imran M Shoaib M. A deep learning model based on concatenation approach for the diagnosis of brain tumor. IEEE Access. (2020) 8:55135–44. doi: 10.1109/ACCESS.2020.2978629

[28] Sultan HH Salem NM Al-Atabany W. Multi-classification of brain tumor images using deep neural network. IEEE Access. (2019) 7:69215–25. doi: 10.1109/ACCESS.2019.2919122

[29] Swati ZNK Zhao Q Kabir M Ali F Ali Z Ahmed S. Content-based brain tumor retrieval for MR images using transfer learning. IEEE Access. (2019) 7:17809–22. doi: 10.1109/ACCESS.2019.2892455

[30] Wang G Li W Zuluaga MA Pratt R Patel PA Aertsen M. Interactive medical image segmentation using deep learning with image-specific fine tuning. IEEE Trans Med Imaging. (2018) 37:1562–73. doi: 10.1109/TMI.2018.2791721, PMID: 29969407 PMC6051485

[31] Rehman A Naz S Razzak MI Akram F Imran M. A deep learning-based framework for automatic brain tumors classification using transfer learning. Circuits, Systems, and Signal Processing. (2020) 39:757–75. doi: 10.1007/s00034-019-01246-3

[32] He K Zhang X Ren S Sun J. Deep residual learning for image recognition. 2016 IEEE Conference on Computer Vision and Pattern Recognition (CVPR). Las Vegas, NV, USA. (2016). p. 770–8.

[33] Lee C. Y. Xie S. Gallagher P. Zhang Z. Tu Z. (2015). Deeply-supervised with intelligence and statistics. In: Guy L SVN Vishwanathan, eds. Proceedings of the Eighteenth International Conference on Artificial Intelligence and Statistics. Vol. 38, San Diego, California, USA: PMLR. (2015). p. 562–70.

[34] Milletari F Navab N Ahmadi SA. V-net: fully convolutional neural networks for volumetric medical image segmentation In: 2016 fourth international conference on 3D vision (3DV). Stanford, CA, USA: IEEE. (2016). 565–71.

[35] Chen J Lu Y Yu Q Luo X Adeli E Wang Y. TransUNet: transformers make strong encoders for medical image segmentation. arXiv. (2021) 2021:66–76. doi: 10.48550/arXiv.2102.04306

[36] Sahayam S Nenavath R Jayaraman U Prakash S. Brain tumor segmentation using a hybrid multi-resolution U-Net with residual dual attention and deep supervision on MR images. Biomedical Signal Processing Control. (2022) 78:103939. doi: 10.1016/j.bspc.2022.103939

[37] Wang J Zhang X Guo L Shi C Tamura S. Multi-scale attention and deep supervision-based 3D UNet for automatic liver segmentation from CT. Math Biosci Eng. (2023) 20:1297–316. doi: 10.3934/mbe.2023059, PMID: 36650812

[38] Ma S Tang J Guo F. Multi-task deep supervision on attention R2U-net for brain tumor segmentation. Front Oncol. (2021) 11:704850. doi: 10.3389/fonc.2021.704850, PMID: 34604039 PMC8484917

[39] Cui J. Xiao R. Fang S. Pei M. Yu Y. (2022). Encoding feature supervised UNet++: redesigning supervision for liver and tumor segmentation. arXiv preprint arXiv:2211.08146. doi: 10.48550/arXiv.2211.08146

[40] Huang L. Miron A. Hone K. Li Y. (2024). Segmenting medical images: from UNet to res-UNet and nnUNet. 2024 IEEE 37th International Symposium on Computer-Based Medical Systems (CBMS). Guadalajara, Mexico and Los Alamitos, CA, USA: IEEE Computer Society. arXiv preprint arXiv:483–9.

[41] Rahman M. Ali S. Khan A. (2025). Enhancing Brain Tumor Segmentation Using Channel Attention and Transfer learning. arXiv preprint arXiv:2501.11196. doi: 10.48550/arXiv.2501.11196

[42] Liu B. Dolz J. Goldman A. Kobbi R. Ayed I. The hidden label-marginal biases of segmentation losses. CoRR (2021) arXiv:2104.08717.

